# Neural Plasticity and Proliferation in the Generation of Antidepressant Effects: Hippocampal Implication

**DOI:** 10.1155/2013/537265

**Published:** 2013-06-19

**Authors:** Fuencisla Pilar-Cuéllar, Rebeca Vidal, Alvaro Díaz, Elena Castro, Severiano dos Anjos, Jesús Pascual-Brazo, Raquel Linge, Veronica Vargas, Helena Blanco, Beatriz Martínez-Villayandre, Ángel Pazos, Elsa M. Valdizán

**Affiliations:** ^1^Departamento de Fisiología y Farmacología, Instituto de Biomedicina y Biotecnología de Cantabria (IBBTEC), Universidad de Cantabria-CSIC-IDICAN, Santander, Cantabria, Spain; ^2^Centro de Investigación Biomédica en Red de SaludMental (CIBERSAM), Instituto de Salud Carlos III, Santander, Cantabria, Spain; ^3^Stem Center, Clínica Palmaplanas, Camí dels Reis 308, Palma de Mallorca, Spain; ^4^The Research Group for Neurobiology and Gene Therapy, KU Leuven, Leuven, Belgium; ^5^Department of Physiology and Pharmacology, School of Medicine, Cardenal Herrera Oria s/n, University of Cantabria 39011 Santander, Spain

## Abstract

It is widely accepted that changes underlying depression and antidepressant-like effects involve not only alterations in the levels of neurotransmitters as monoamines and their receptors in the brain, but also structural and functional changes far beyond. During the last two decades, emerging theories are providing new explanations about the neurobiology of depression and the mechanism of action of antidepressant strategies based on cellular changes at the CNS level. The neurotrophic/plasticity hypothesis of depression, proposed more than a decade ago, is now supported by multiple basic and clinical studies focused on the role of intracellular-signalling cascades that govern neural proliferation and plasticity. Herein, we review the state-of-the-art of the changes in these signalling pathways which appear to underlie both depressive disorders and antidepressant actions. We will especially focus on the hippocampal cellularity and plasticity modulation by serotonin, trophic factors as brain-derived neurotrophic factor (BDNF), and vascular endothelial growth factor (VEGF) through intracellular signalling pathways—cAMP, Wnt/**β**-catenin, and mTOR. Connecting the classic monoaminergic hypothesis with proliferation/neuroplasticity-related evidence is an appealing and comprehensive attempt for improving our knowledge about the neurobiological events leading to depression and associated to antidepressant therapies.

## 1. Introduction

Major depressive disorder (MDD) constitutes the first leading cause of years lived with disability [1], and its incidence is on the rise globally. Yet, until recently, little was known about its pathogenesis, as these conditions are not associated with relevant brain alterations or clear animal models for spontaneous recurrent mood episodes.

The clinical phenomenology of major depression implicates brain neurotransmitter systems involved in the regulation of mood, anxiety, fear, reward processing, attention, motivation, stress responses, social interaction, and neurovegetative function [2]. MDD is associated with blunted reactivity to both positively and negatively stimuli [3]; thus, the decline in hedonic responses may be related to generalized affective insensitivity, instead of deficits in the capacity to feel pleasure at the level of basic sensory experience [4]. From the middle of the last century, a great effort has been made to elucidate the brain areas involved in emotion control and in the pathophysiology of mood disorders. Animal and human studies have indicated the involvement of the limbic system—including the hippocampal formation, cingulate gyrus, and anterior thalamus—the amygdala and different cortical structures as well as the hypothalamus in these processes [5, 6]. These structures are connected in two main networks: the “orbital” and the “medial prefrontal networks.” The orbital network appears to function both as a system for integration of multimodal stimuli and as a system for assessment of the value of those stimuli, and, probably, the support of abstract assessment of reward. The medial network is probably more significant for mood disorders [7].

In depressed subjects, the structures of the medial prefrontal network have been shown to contain alterations in gray matter volume, cellular elements, neurophysiological activity, receptor pharmacology, and gene expression. Dysfunction within this system underlies the disturbances in emotional behavior and other cognitive aspects of the major depressive disorder. Treatments for depression, involving pharmacological, neurosurgical, and deep brain stimulation methods, appear to suppress pathological activity within the components of medial prefrontal network such as the subgenual anterior cingulate cortex, ventromedial frontal cortex, striatum, and amygdala [6].

Although the causes of MDD are not yet completely known, genetic factors appear to play an important role although other factors deal with acute or chronic stress, childhood trauma, viral infections, and others [12, 13]. Regarding genetic causes, certain polymorphisms in genes related to the serotonergic system as the serotonin transporter, the brain-derived neurotrophic factor, the monoamine oxidase A, or the tryptophan hydroxylase 1, may increase the risk for depression or the vulnerability to stress [14]. Not all the studies published to date have found gene-environment interactions; however, the combination of both factors seems to predict more accurately a person's risk to suffer from major depressive disorder better than genes or environment alone.

The discovery that some drugs as iproniazid and imipramine exert an antidepressant effect dates back to the 1950s [15]. In 1965, it was shown that these drugs act through the monoaminergic system by increasing the brain levels of those monoamines [16]. These observations led to the development of the classical “monoaminergic hypothesis of depression,” which proposes that low monoamine brain levels in depressed individuals are responsible for this pathology. The classic antidepressants that increase monoamine neurotransmitters in the synaptic cleft are generally used for first-line treatment. However, the clinical benefit of these treatments is not immediate, taking 3-4 weeks to obtain a full response. Other therapeutic problems of currently used antidepressant drugs include relapses, drug side effects, incomplete resolution, residual symptoms, and drug resistance.

Traditionally, research in the neurobiology of major depressive disorder has been focused on monoamines. However, several lines of evidence [17] have led to the conclusion that the abnormalities associated to depression go beyond monoaminergic neurotransmission: thus, the development of better antidepressants will surely depend on the discovery and understanding of new cellular targets. In this regard, in the late 90's a new hypothesis has tried to explain major depression based on molecular mechanisms of neuroplasticity [18].

The “neuroplasticity hypothesis” was postulated based on several findings: first, stress decrease hippocampal neurogenesis and synaptic plasticity in prefrontal cortex (PFCx) [19–22]. Moreover, most known antidepressant therapies stimulate the proliferation of hippocampal progenitor cells, which constitutes the first stage of adult hippocampal neurogenesis [23]. However, the contribution of hippocampal neurogenesis to the pathogenesis of depression is far from being fully understood. Second, hippocampal morphologic analyses in depressed patients reveal volume loss and gray matter alterations. While some studies suggest that decreased adult neurogenesis could be responsible for such fluctuating changes, others show that the hippocampal volumetric reductions could be due to changes in neuropil, glial number, and/or dendritic complexity, and not necessarily to a cell proliferation decrease [24]. Third, different neuroplasticity- and proliferation-related intracellular pathways appear to be involved in the antidepressants' action as brain-derived neurotrophic factor (BDNF) [25], *β*-catenin [10, 26], or the mammalian target of rapamycin (mTOR) [27].

## 2. Cell Proliferation and Plasticity Role in Mood Disorders and Antidepressant Treatments

Dentate gyrus proliferation is decreased by stress [19–22], and in several animal models of depression as unpredictable stress, chronic administration of corticosterone, olfactory bulbectomy, or maternal deprivation [22, 28–31]. This loss in cell proliferation is correlated with a decreased hippocampal volume [32–34]. Hippocampal proliferation decrease is also observed in other disease models such as diabetic mice, which present a high incidence of depression reported in individuals with that primary illness [35]. In these animals, the reduced hippocampal proliferation is reversed with chronic antidepressant treatments [35]. In animals subjected to acute or chronic stress, a period of at least 24 h or 3 weeks, respectively, is required to get a recovery of the cellular proliferation [29]. However, although all these changes have been extensively studied, major depression is not generally considered as “hippocampal disorder.”

It is unlikely that impaired adult hippocampal neurogenesis alone may fully explain the neuropathology of major depression. In this sense, other studies have addressed cellular proliferation in anatomical structures quite relevant to depressive disorders, such as prefrontal cortex and amygdala, by using animal models of depression. Thus, medial frontal cortex presents a reduction in cell proliferation [36], downregulation of genes implicated in cell proliferation [37, 38], decreased cell growth and survival, and apoptosis inhibition [38]. Structures such as amygdala present an opposite pattern, with an increase in neuronal dendrite length in stress models [39]. Chronic administration of antidepressants leads to an increased proliferation in prefrontal cortex [36, 40] although the fate of the new generated cells goes toward the formation of glia rather than neurons, in contrast to hippocampus [36]. No data are available regarding antidepressant effects on amygdalar cell proliferation; however, this structure has been involved in the negative control of the hippocampal cell survival induced by antidepressant treatments, based on the increased cell survival observed in hippocampus, after the basolateral complex of the amygdale (BLA) lesion [41]. It is interesting to note that amygdala—implicated in fear-related learning that impairs the memory processing of the Hp-PFCx memory—shows an enhancement of LTP under stress situations, which is not reverted by antidepressants. Thus, antidepressants as tianeptine are able to restore the normal functionality of Hp and PFCx under stress situations, while the amygdala retains the ability to increase its activity in the same stress conditions [42].

The disturbed adult hippocampal neurogenesis cannot fully explain major depression. It could only be the most conspicuous feature of a more fundamental type of cellular plasticity, which could also govern the prefrontal cortex and other regions. It has also been proposed that, in addition to neural proliferation, changes in synaptic plasticity would also be involved in the biological basis of depression [19], being modulated by antidepressant treatments [43, 44]. PFCx is also a region sensitive to stress-induced effects, with a reduction in the number and length of spines [45] in apical dendrites of the pyramidal cells in the medial prefrontal cortex area [46, 47], as well as changes in the number, morphology, metabolism, and function of glial cells, that produce changes in the glutamatergic transmission, resulting in memory impairments [48–51], and reduced synaptic plasticity in the Hp-PFCx neuronal pathway [52]. The increase in extracellular glutamate could be one of the reasons underlying the molecular changes associated to stress [51]. However, while frontal cortex and hippocampus are reduced and hypofunctional in major depression, structures such as amygdala present hypertrophy and hyperactivity. Changes in synaptic plasticity will reflect a vulnerability to suffer a depression episode [39].

An increased apoptosis has also been related to a higher risk of suffering major depression since increased cell death in areas as dentate gyrus (DG), CA1, and CA4 areas of the hippocampus, entorhinal cortex, and subiculum are reported in studies using human postmortem brain samples though this phenomenon does not seem to account for the hippocampal volume reduction [53]. Animal studies also report that acute stress increase hippocampal apoptosis [29], while chronic stress induces no changes [29], increased apoptosis in cortex [54, 55], or hippocampus [55]. Antidepressant treatment decreases cell death by different mechanisms, as the activation of the expression of trophic factors (BDNF and its receptor TrkB) which results in increased cell survival [56, 57] or directly reducing cellular apoptosis in animal stress models as reported for fluoxetine [28, 58].

**Figure 1:**
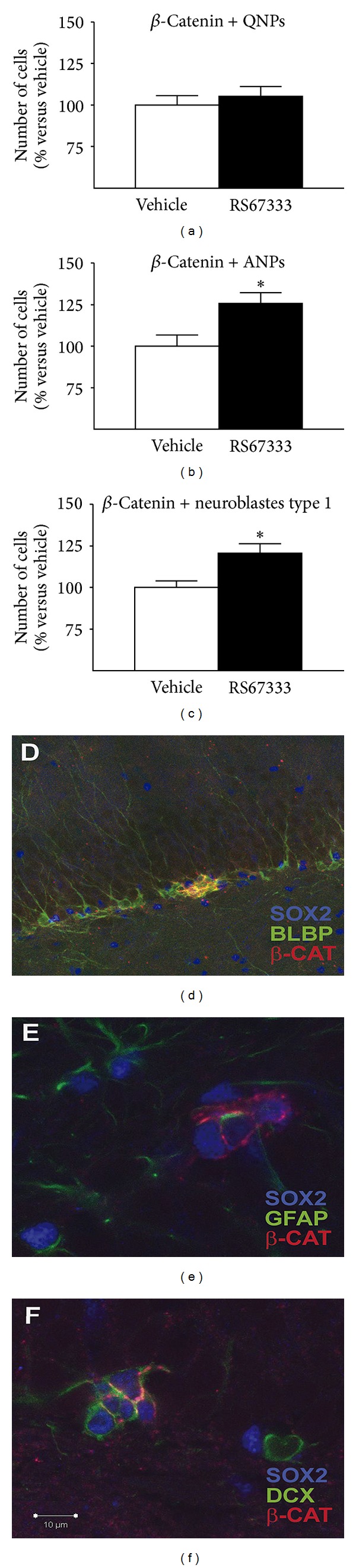
RS67333 increases the number of ANPs (b) and neuroblasts type 1 (c) that express *β*-catenin, but not total number of QNPs cells (a). Photomicrographs illustrating *β*-catenin expression in neural progenitors (d), ANPs (e), and neuroblasts (f). The results are the Mean ± S.E.M. *P < 0.05 versus vehicle. Bar: 10 *μ*M, modified from Pascual-Brazo et al., 2012 [8].

It has been suggested that an increase in serotonin levels mediates the raise in cell proliferation, while the depletion of this neurotransmitter does not lead to an immediate effect over the hippocampal cell division [59]. In line with that, the direct or indirect modulation leads to an increase in proliferation. Treatments exerting a direct action over the serotonergic system include chronic but not acute administration with drugs such as tricyclic antidepressants, monoamine oxidase inhibitors (MAOI), serotonin-selective reuptake inhibitors (SSRI), serotonin and noradrenaline reuptake inhibitors (SNRI), and 5HT_4_ agonists [8, 19, 20, 60–65]. A nonpharmacological intervention such as the silencing of the serotonin transporter (SERT) by RNAi in dorsal raphe serotonergic neurons also leads to increased hippocampal proliferation [66]. The administration of other drugs, such as lithium in combination with antidepressants as desipramine, produces an increase in hippocampal proliferation [67] and a decrease in apoptosis of hippocampal progenitor cells in irradiated animals [68]. Treatments with antidepressants that increase serotonin levels in brain act by targeting different progenitor cell populations. Thus, chronic administration of the SSRI fluoxetine [69] or subchronic treatment with a 5-HT_4_ agonist [8] increases cell proliferation and neurogenesis-targeting amplifying neural progenitors (ANPs) (Figure 1) while chronic electroconvulsive seizure (ECS) produces a fast-acting effect targeting both quiescent neural progenitors (QNPs) and ANPs [70]. An increased hippocampal proliferation as a consequence of chronic antidepressant treatment has been proven necessary for some [71–73], but not all the antidepressant-like effects in animals. The antidepressant-like effects have been related to the increased hippocampal proliferation [8, 71, 74], dendritic arborization, maturation, and functional integration of newborn neurons [75]. However, other drugs with potential antidepressant action do not mediate their effect through the activation of progenitor cells division, since the complete elimination of hippocampal proliferation by direct irradiation of this structure does not block the antidepressant response promoted by the blockade of drugs acting on other neurotransmitter systems as the corticotrophin-releasing factor receptor (CRF) or arginine vasopressin 1b (V1b) receptors [76].

## 3. Pathways Leading to Proliferation and Neural Plasticity Changes That Exert Antidepressant-Like Effect

Classically, the modulation of different neurotransmitter systems has been implicated in the mediation of the antidepressant effects, and, for some of them, a link with proliferative or plastic changes has been reported. The traditionally involved neurotransmitter systems include the serotonergic, adrenergic, and dopaminergic ones, while others, such as the glutamatergic and cannabinoid systems and the corticotropin-releasing factor (CRF) system implicated in the secretion of ACTH are acquiring increasing importance in the last years. Here we will focus on the serotonergic receptors most relevant to modulating neural proliferation and synaptic plasticity processes.

### 3.1. Serotonergic Receptors

Serotonin has a positive role in the regulation of hippocampal neurogenesis. The partial lesion of dorsal and medial raphe nuclei, which results in a decrease of serotonergic neurons that innervate the dentate gyrus of the hippocampus and other projection areas as cortex and amygdala, decreases the proliferation in the subgranular zone of the dentate gyrus [132]. Several serotonin receptors have been involved in the antidepressant-induced increase of cell proliferation in the hippocampus, together with neurite outgrowth and cell survival in cells expressing these receptors [80]. However, other authors report a lack of changes in proliferation and/or neurotrophic factors expression after chronic treatment with the antidepressant fluoxetine, questioning the importance of the serotonergic system in hippocampal proliferation [133–135].

#### 3.1.1. 5-HT_1A_ Receptors

The importance of this serotonergic subtype in the effects of antidepressants has been shown in studies in vivo using 3 day treatment with the 5-HT_1A_ agonist 8-OH-DPAT [77], and chronic administration of this drug [74, 79, 80] that produce an increase in proliferation in the subgranular zone (SGZ) of the hippocampus that depends on the 5-HT_1A_ postsynaptic receptors. In studies using hippocampal neural progenitors, the serotonin-mediated increase in proliferation is blocked by the 5-HT_1A_ antagonist WAY100,635 [78]. On the contrary, the acute administration of 5-HT_1A_ antagonists produces a decrease of hippocampal proliferation [136], or no changes after 14 days [79]. Knock out animals for the 5-HT_1A_ receptor subtype present no changes in basal proliferation compared to wild type animals [9, 71], but present a decreased hippocampal cell survival [9]. The 5-HT_1A_ receptor subtype has been proven necessary for the hippocampal proliferative effect of some antidepressants as fluoxetine [71], although other drugs as imipramine, acting on other neurotransmitter systems, increases hippocampal proliferation in a 5-HT_1A_-independent manner [71] (Table 1).

#### 3.1.2. 5-HT_2A/C_ Receptors

The role of the 5-HT_2A/C_ receptors in the regulation of neurogenesis is less clear. The chronic administration of 5-HT_2A_ antagonists as ketanserin [81], and 5-HT_2C_ antagonists as SB243,213 and S32006 [82], produce the increase in hippocampal proliferation, while the acute treatment with 5-HT_2A/C_ agonists or antagonists produce no changes or a decrease in proliferation, respectively [74, 81]. Interestingly, the subchronic treatment with ketanserine in combination with the SSRI fluoxetine increases a series of synaptic plasticity markers as *β*-catenin and N-cadherin present in the membrane fraction, together with BDNF gene expression, however, hippocampal proliferation is not significantly modified [83]. The increased proliferation or synaptic plasticity parallel the antidepressant-like effect observed for the treatments with antagonists [83, 137, 138], while the administration of 5-HT_2A_ agonist counteracts the effect of SSRIs [137]. The blockade of 5-HT_2A_ receptor subtype located in GABAergic interneurons produces the activation of hippocampal pyramidal neurons [139] that modulates dendritic activation and synaptic plasticity [140] (Table 1).

#### 3.1.3. 5-HT_4_ Receptors

In the last years, the 5-HT_4_ receptor subtype has been proven to have an outstanding role on the depressive pathology. This receptor subtype density and signaling cascade through cAMP are up-regulated in the frontal cortex and caudate-putamen of depressed humans [141]. Chronic treatments with classical antidepressants produce a desensitization of this subtype in structures as hippocampus [97, 98]. In the last years, it has been described a short-term antidepressant-like response mediated by 5-HT_4_ partial agonists [8, 64, 65], or when coadministered with classical antidepressants [63]. The antidepressant effect of 5-HT_4_ agonist is mediated by an increase in hippocampal proliferation in vivo [8, 64], together with other proliferative and plasticity markers as *β*-catenin, Akt [8], BDNF [8, 65], phosphorylated cAMP response element binding (CREB) protein [8, 63–65]. The 5-HT_4_ implication in serotonin-induced hippocampal proliferation has been observed blocking this receptor with the 5-HT_4_ antagonist DAU 6285 in primary hippocampal progenitor cell cultures [78] (Table 1).

#### 3.1.4. 5-HT_6_ Receptors

The role of the 5-HT_6_ receptor subtype in depression is not clear, but tricyclic antidepressants as amitriptyline and atypical antidepressants as mianserin have high affinity for this serotonin receptor subtype, acting as antagonists [142]. Moreover, the expression of the 5-HT_6_ receptors is regulated by glucocorticoid levels [142]. This receptor subtype is present postsynaptically in brain areas as cortex and hippocampus and is implicated in learning and memory [142]. The action over this receptor to date is contradictory since it has been published that both antagonists and agonists exert antidepressant and anxiolytic effects alone [142, 143] or enhance the beneficial effect when combined with antidepressant drugs [144]. When locally administered on hippocampus, 5-HT_6_ antagonists produce antidepressant-like effect [145]. However, this effect is not mediated by increased neurogenesis but for an increase in neural cell adhesion molecule polysialylation (PSA-NCAM) that may mediate memory consolidation [84] through long-term changes in synaptic plasticity [146] (Table 1).

#### 3.1.5. 5-HT_7_ Receptors

The 5-HT_7_ receptor subtype is also involved in the antidepressant effect. Recent studies have shown that the blockade of the 5-HT_7_ receptor subtype produces antidepressant-like behaviour [147, 148]. This is supported by studies in animal depression models as the olfactory bulbectomy [85], the antidepressant-like behaviour of knock-out mice for the 5-HT_7_ receptor subtype [149], and clinical data using the antagonist Lu AA21004 [150]. Moreover, a 7-day treatment with the 5-HT_7_ antagonist SB-269970 produces an increase in proliferation in the subgranular zone of the hippocampus [85] although changes in the number of dividing cells do not appear in 5-HT_7_ knock-out animals [86] (Table 1).

### 3.2. Neurotrophic Factors

In an attempt to explain those brain changes implicated on depression and/or antidepressant effect that could not be included in the initial monoaminergic hypothesis of depression, it was postulated the so-called “Neurotrophic hypothesis of Depression” that later was revised to a “new” “hypothesis of neuroplasticity” [18]. This hypothesis links the changes in depression models to a decrease of brain-derived neurotrophic factor (BDNF) and the antidepressant effect to an increase in BDNF in hippocampus [18, 19, 151, 152]. Moreover, the decreased BDNF observed in heterozygous knock-out mice (BDNF^+/−^) is related to a depression-like phenotype [18]. These changes in brain BDNF expression are paralleled by serum levels, so that it has been proposed as a biomarker for depression disease, positive or negative response of the individuals to the antidepressant treatment [153–156], and even a marker of suicidal depression [157]. However, the role of BDNF is still not clear in the depressive pathology since some authors describe a lack of changes on the BDNF levels associated to stress animal models [57, 158–160].

The infusion of BDNF in brain [161, 162] or more specifically in hippocampus [163, 164] produces antidepressant-like effects. Moreover, within the hippocampus, the infusion of BDNF in the DG but not in the CA1 region produces an antidepressant-like effect [163], which is supported by the lack of antidepressant action in mice selectively knocked out for the BDNF gene in the DG and not in the CA1 [160]. Even peripherally administered BDNF is able to display antidepressant-like actions [165], resembling the increased serum BDNF observed after antidepressant treatments [166]. 

**Figure 2:**
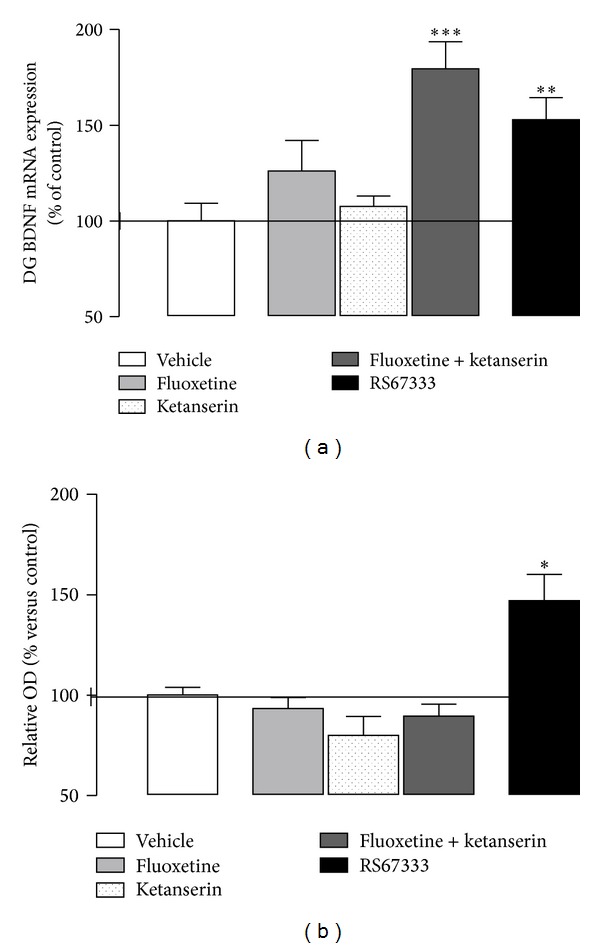
BDNF mRNA expression (a) and protein level (b) in the dentate gyrus of the hippocampus (DG) or total hippocampus, respectively, after 7-day treatment with the 5-HT_4_ partial agonist RS67333 (1.5 mg/kg/day) (modified from [8]) and 7-day coadministration of the SSRI fluoxetine (5 mg/kg/day) and the 5-HT_2A_ antagonist ketanserin (0.1 mg/kg/day). *P < 0.05 versus vehicle. Modified from Pascual-Brazo et al., 2012 [8], and Pilar-Cuéllar et al., 2012 [83].

Chronic administration of antidepressants produces an increase in hippocampal BDNF mRNA expression and BDNF protein levels (Figure 2) [8, 65, 167]. The blockade of 5-HT_2A_ receptor reverses the effect of stress-induced downregulation of BDNF mRNA expression in hippocampus [168]. Also, the subchronic treatment with SSRI and 5-HT_2A_ antagonists is able to increase BDNF expression in the dentate gyrus of the hippocampus; however, the protein level is not yet modified in subchronic treatments (Figure 2) [83].

The main role of BDNF regarding adult neurogenesis is not linked to proliferation, but to the increase in cell survival, as described using BDNF and its receptor TrkB knock-out animals which present a reduced BDNF expression [61, 169]. BDNF is implicated in synaptic plasticity, and proteins as neuritin that are induced by BDNF are decreased in stress-induced animal models of depression [170] and increased after chronic antidepressant treatment, contributing to the BDNF antidepressant effect [170, 171].

The existence of a single-nucleotide polymorphism (SNP) in the human BDNF gene, BDNF (Val66Met) is associated to reduced BDNF secretion [172], and to an increased incidence of neuropsychiatric disorders [173, 174]. In animals BDNF (Val66Met) predisposes to a depression-like behaviour after stress situations that recover normal values after the administration of antidepressants [175]. This polymorphism is also associated to nonresponders after antidepressant treatment [176].

Other important trophic factor is the vascular endothelial growth factor (VEGF) implicated in the “vascular niche hypothesis of adult neurogenesis.” This theory proposes the need of vascular recruitment associated to active sites of neurogenesis formed by proliferative cells that present an endothelial phenotype in 37% of the cases [177]. VEGF expression is reduced in hippocampal dentate gyrus after irradiation [178], and in stress models [179] although other authors do not show changes associated to stressed animal models [180]. From studies using irradiated rats, it was proposed that the decrease of progenitor cells responsible for the expression of VEGF would underlie the decrease of this factor [178].

Some antidepressant treatments, as the electroconvulsive therapy (ECS) [178, 181, 182], approache with antidepressant-like effect as exercise [180], or mood stabilizers as lamotrigine [183], result in the upregulation of VEGF expression. Moreover, the local administration of this trophic factor produces an increase in hippocampal proliferation [178]. In addition, the silencing of hippocampal VEGF [184] or the use of antagonists for its receptor Flk-1 [180] blocks its antidepressant-like effect and decreases markers of newborn neurons as doublecortin (DCX).

Even though these data indicate the importance of VEGF brain levels in the depressive disorder, preliminary reports do not show a clear correlation between peripheral VEGF and depressive disorders, not allowing for the use of this molecule as a marker of depression and/or antidepressant response [185, 186].

The activation of receptor tyrosine kinases by neurotrophic factors promotes the activation of the PI3K/Akt pathway that is linked to the Wnt/*β*-catenin through the inhibition of GSK-3*β* and to the mTOR pathway through the phosphorylation of mTOR protein [187] that are discussed below. The PI3K/Akt pathway per se has an outstanding role in promoting adult hippocampal proliferation and the inhibition of cell differentiation [188]. Antidepressant treatments also produce increases in Akt levels in structures as hippocampus [8, 10] and frontal cortex [27].

### 3.3. Intracellular Pathways

#### 3.3.1. Cyclic Adenosine Monophosphate (cAMP) Cascade

The upstream and downstream components of the cAMP signaling pathway have been extensively involved in the pathophysiology of mood disorders as well as in the actions of antidepressant drugs. Alterations in several elements of this pathway, such as G proteins (Gs or Gi), adenylate cyclase (AC), cAMP levels, cAMP-dependent protein kinase (PKA), and the cAMP response element-binding protein (CREB) transcription factor, have been described in peripheral cells and the postmortem brain of patients with affective disorders, both untreated or after antidepressant therapy [11, 100, 189, 190]. Various elements along this pathway have been identified as potential targets for antidepressant drugs (Table 2).

**Figure 3:**
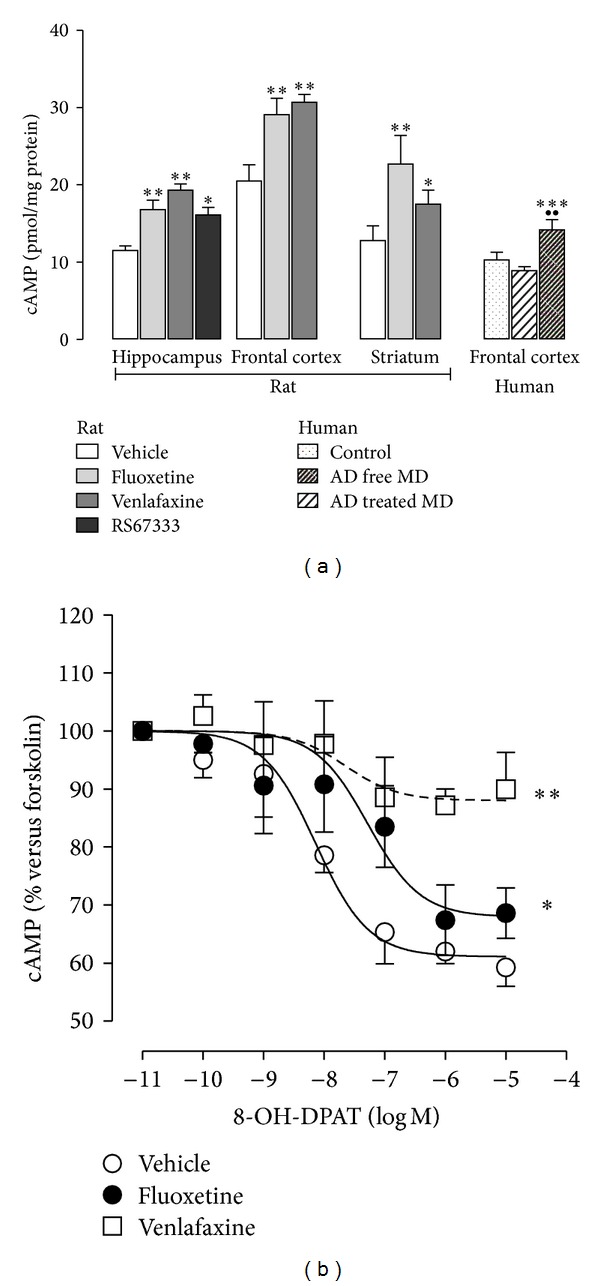
Antidepressant treatment increases basal cAMP in both rat and human. (a) Increase of basal cAMP levels in rat hippocampus, frontal cortex and striatum after chronic (14 days) antidepressant treatment with fluoxetine (10 mg/kg/day) and venlafaxine (40 mg/kg/day), and subchronic treatment (3 days) with the 5HT_4_ agonist RS67333 (1.5 mg/kg/day) and in postmortem frontal cortex samples from control, antidepressant free-depressed subjects (AD-free MD) and antidepressant-treated depressed subjects (AD-treated MD). cAMP is expressed in pmoles/mg protein. *P < 0.05; **P < 0.01 and ***P < 0.001 versus vehicle or control subjects; ^●●^ *P* < 0.01 versus antidepressant-free depressed subjects. (b) Modulation of 5-HT_1A_ receptor subtype-mediated inhibition of cAMP accumulation by antidepressant drugs. Chronic antidepressant treatment with fluoxetine (10 mg/kg/day) and venlafaxine (40 mg/kg/day) for 14 days downregulates 8-OH-DPAT inhibition of forskolin-induced cAMP accumulation. (a) Modified from Mostany et al., 2008 [10], Pascual-Brazo et al., 2012 [8], and unpublished results and (b) unpublished results.

In peripheral cells and postmortem brains of patient with mayor depression, there is a reduction of the adenylyl cyclase (AC) activity in response to forskolin [87], *β*_2_-adrenergic agonists [88–93], and *α*2-adrenoceptor agonists [94]. Chronic treatment with antidepressant drugs produces the increase in cAMP levels in rat hippocampus, cortex, and striatum, as well as in postmortem human frontal cortex samples from depressed patients (Figure 3(a); personal observation). This effect has been attributed to both enhanced coupling of Gs proteins to adenylyl cyclase and increased adenylyl cyclase activity [95, 96]. The direct injection of cAMP or inhibition of cAMP degradation by rolipram produces antidepressant-like effect in animals [99]. Chronic antidepressant treatment in rat desensitizes cAMP response to serotonergic receptor as 5-HT_1A_ receptor (Figure 3(b)) and 5-HT_4_ receptor [8, 97, 98] and increases the CB1-mediated inhibition of adenylyl cyclase (AC) in prefrontal cortex, an effect that is modulated by 5-HT_1A_ receptors [100].

The next step in this signaling pathway is the activation of cAMP-dependent protein kinase (PKA) by cAMP, so that PKA activity is increased after chronic antidepressant administration [101].Active PKA phosphorylates proteins as CREB, a transcription factor that regulates the expression of several genes involved in neuroplasticity, cell survival, and cognition [191–195].

**Figure 4:**
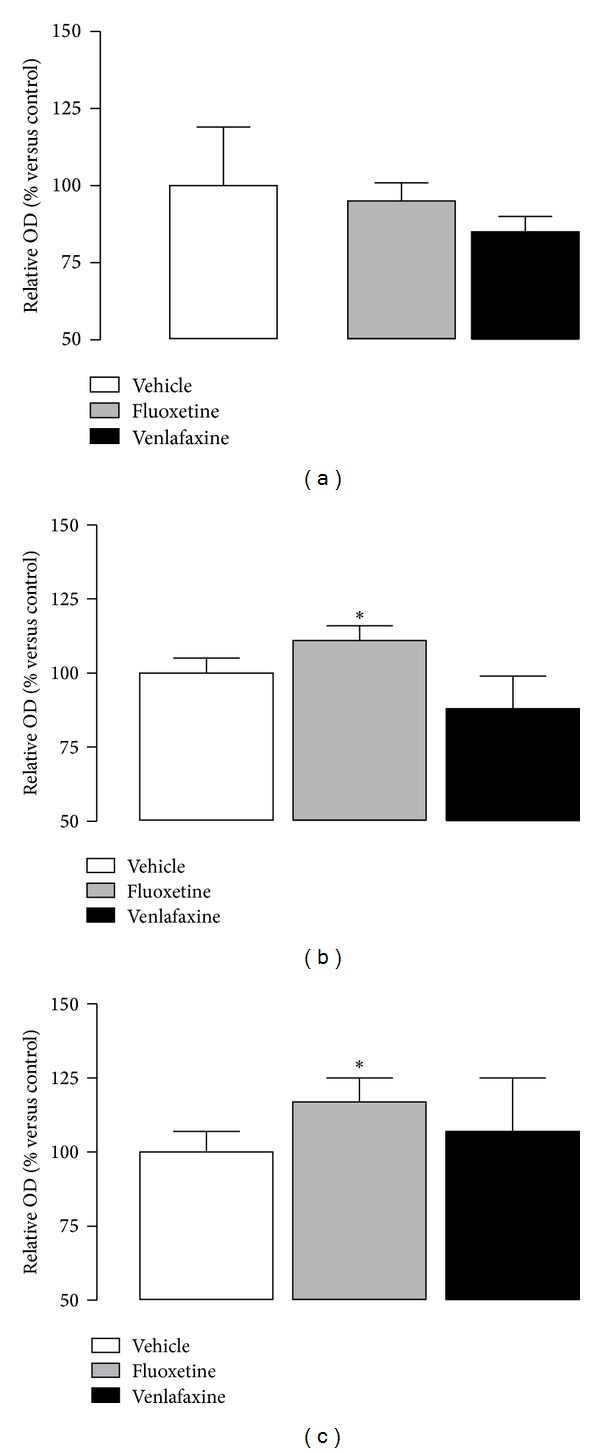
Western blot analyses of hippocampal CREB (a) and p-CREB (b) levels and ratio p-CREB/CREB (c) after chronic antidepressant treatment (14 days) with fluoxetine (10 mg/kg/day) and venlafaxine (40 mg/kg/day). Note that p-CREB levels and ratio p-CREB/CREB are increased after fluoxetine treatment, but not total CREB, or after venlafaxine treatment in total cell lysate from hippocampus of rats. Values are means ± S.E.M. Corresponding to densitometry levels of the proteins expressed as the percentage of the same proteins in vehicle-treated animals. *P < 0.05 versus vehicle. Modified from Mostany et al., 2008 [10], and Mato et al., 2010 [100].

CREB has been widely involved in the pathophysiology of depression and both behavioural and cellular responses to antidepressant treatments [11, 190]. Hippocampal expression of CREB is reduced in response to stress exposure [102, 103]. In contrast, chronic but not acute antidepressant therapy and electroconvulsive shock (ECS) increase levels of CREB mRNA, CREB protein (Figure 4), and CREB activity—promoting the phosphorylation of this protein—effects that seems to be area and drug dependent [11, 103, 105–108]. Thus, increased phosphorylated CREB levels in hippocampus are linked to antidepressant-like behaviour [44], as observed after viral-mediated overexpression of CREB in hippocampus in behavioural models of depression [44]. Contrary to what could be expected, CREB overexpression in the nucleus accumbens produces prodepressive effects [111], and lowered CREB in the nucleus accumbens in mice exhibits an antidepressant-like response [196]. A different pattern appears also for amygdala, in which high CREB levels produce opposite effects depending on the timing. Thus, when CREB overexpression is induced before learned helplessness training, there is a prodepressant effect, while the increase of CREB after the training is antidepressant [109]. Studies in postmortem human brain indicate lower levels of CREB protein in depressed antidepressant-free subjects, in contrast to the increased CREB level in patients taking an antidepressant at the time of death [104]. These results are parallel to studies in human fibroblasts of patients with major depression [110], which is consistent with animal studies. Among the several target genes regulated by CREB, two of the more relevant, are the brain-derived neurotrophic factor (BDNF) and the vascular endothelium growth factor (VEGF) [19, 60, 197, 198].

A growing body of data shows that other signalling cascades can modulate CREB activity through phosphorylation, such as the calcium/calmodulin-dependent kinase (CaMKII) and the mitogen-activated protein (MAP) kinase cascades, and may also be implicated in the mechanism of action of antidepressants [11, 199].

Initially, all effects of cAMP increase were attributed to the activation of PKA/CREB, but two novel targets as the cAMP-regulated ion channels and Epac (exchange protein directly activated by cAMP) are now known to be involved in mediating cAMP responses. An increase in Epac-2 levels, but not Epac-1, has been found in postmortem samples of prefrontal cortex and hippocampus of depressed subjects [112].

#### 3.3.2. Wnt/*β*-Catenin Pathway

The Wingless-type (Wnt) family of proteins has key roles in many fundamental processes during neurodevelopment [200]. The role of this pathway in neural development, through the modulation of neural stem cells' (NSC) proliferation and differentiation, has been clearly demonstrated [201]. Some of the processes regulated by Wnt/*β*-catenin pathway activity are neural differentiation [202], hippocampal formation [203, 204], dendritic morphogenesis [205, 206], axon guidance [207, 208], and synapse formation [209]. Moreover, it also plays an important role in spatial learning [210] and memory, including long-term potentiation (LTP) phenomena [159].

In the absence of Wnt signaling, *β*-catenin function is blocked by a destruction complex consisting of Axin, APC, and GSK-3*β* and CK1a kinases [211], which phosphorylates *β*-catenin for destruction in the proteasome [212, 213]. Wnts act through both canonical and noncanonical signal transduction pathways. Canonical Wnt signaling results in the inhibition of GSK-3*β* which is constitutively active, and the non-phosphorylated *β*-catenin is stabilized in the cytoplasm and translocated to the nucleus, which is essential for canonical Wnt signaling [214]. Once in the nucleus, *β*-catenin forms a complex with the T-cell factor/lymphoid enhancer factor (TCF/LEF) transcription factors, to activate the expression of Wnt target genes. TCF/LEF transcription factors are bound to Groucho, a protein producing repressive effects [214]. Nuclear *β*-catenin promotes the displacement of Groucho and the binding of the histone acetylase cyclic AMP response element-binding protein (CREB), activating the transcription machinery [214, 215]. The noncanonical pathway or *β*-catenin independent is mediated through Rac/Rho (Wnt/PCP) or through calcium (Wnt/Ca^2+^) [201].

In the last years several evidences have implicated Wnt-signaling pathway in the pathophysiology and treatment of mood disorders and other cognitive pathologies. GSK-3*β* and *β*-catenin are regulated either directly or indirectly by lithium, valproate, antidepressants, and antipsychotics [8, 10, 113–117], while GSK-3*β* has also been identified as a target for the treatment of Alzheimer's disease [216] (Table 2).

Postmortem human brain samples from depressed subjects and teenage suicide victims present a dysregulation of Wnt/GSK-3*β* signaling with a decrease in *β*-catenin expression in prefrontal cortex [124]. *β*-catenin knock-out mice with 50–70% decrease of *β*-catenin expression in forebrain regions present an increased immobility time in the tail suspension test indicating a depression-like state, but not in other anxiety tests [125].

The inhibition of GSK-3*β* activity, either pharmacologically [117–119], or through deletion in mouse forebrain, results in an increase in brain *β*-catenin levels, as well as in antidepressant-like effects or decreased anxiety [120], as observed by the direct overexpression of *β*-catenin in mouse brain [126]. GSK-3*β* inhibition by lithium is an important regulator of cell survival related to mood stabilizers [215] and displays antidepressant efficacy [115, 118, 215, 217, 218]. In contrast, GSK-3*β* knockin mice displayed increased susceptibility to stress-induced depressive-like behaviour [121], presenting decreased cell proliferation in the subgranular zone of the dentate gyrus, accompanied by a reduction in VEGF, but not BDNF, and blunted neurogenesis in response to antidepressant treatments [219]. These data support the importance of the Wnt pathway activation and *β*-catenin levels associated to mood disorders and their treatment. In addition, SNP variation in the promoter region of GSK-3*β* plays a protective role in the onset of bipolar illness [122] and increased antidepressant response [123].

Recent studies have identified the Wnt/GSK-3*β*/*β*-catenin-signaling pathway as a key regulator of adult neurogenesis in hippocampus [220, 221] or subventricular zone [222], highlighting the role of GSK-3*β* on neural progenitor homeostasis [200]. Wnt proteins are signaling molecules that are released from hippocampal neural stem cells (NSC) and astrocytes, acting autocrinally to regulate proliferation via Wnt canonical pathway [220, 221].

**Figure 5:**
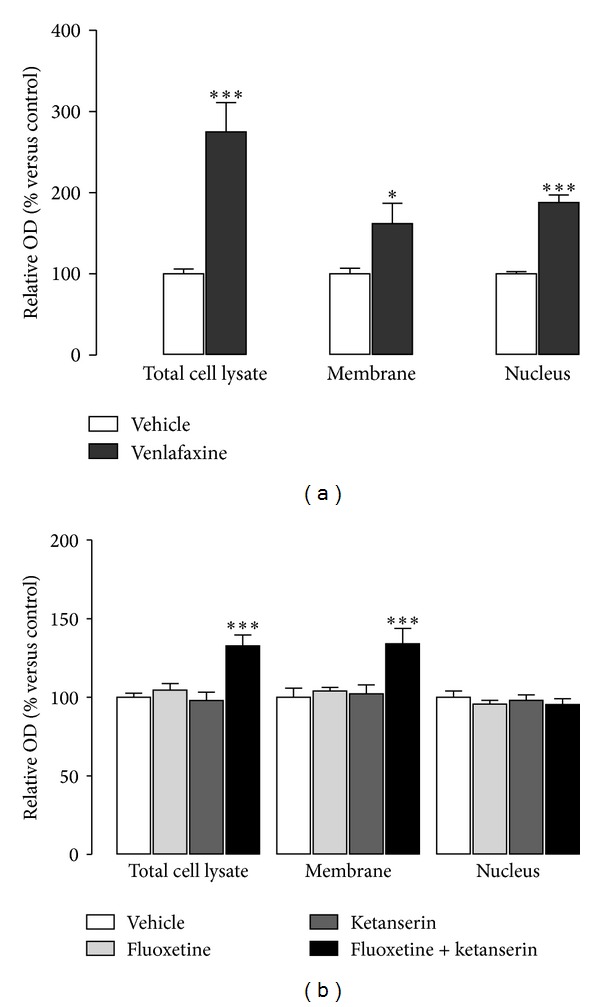
Implication on *β*-catenin subcellular distribution of the chronic (14 days) antidepressant treatment with the SNRI venlafaxine (40 mg/kg/day) (a), and 7-day treatment with the SSRI fluoxetine (5 mg/kg/day), the 5-HT_2A_ antagonist ketanserin (0.1 mg/kg/day), and the combination of both (b). Note that chronic treatment with venlafaxine produces an increase in both membrane-associated and nuclear *β*-catenin, while the subchronic treatment with fluoxetine + ketanserin, only increases *β*-catenin in the membrane but not in the nuclear fraction. *P < 0.05 and ***P < 0.001 versus vehicle. (a) Modified from Mostany et al., 2008 [10], and (b) modified from Pilar-Cuellar et al., 2012 [83].

Wnt/*β*-catenin pathway is activated by antidepressant treatments as electroconvulsive therapy [26], chronic treatments with classical antidepressants as the dual serotonin-noradrenaline reuptake inhibitor (SNRI) venlafaxine (Figure 5(a)) [10], and 5-HT_4_ partial agonists [8]. The antidepressant-induced *β*-catenin increase is observed in the subgranular zone (SGZ) of the dentate gyrus (DG) of the hippocampus, in membrane and nuclear fractions [10, 26]. The increased proliferation observed in SGZ after chronic antidepressant treatments is localized in cell clusters that also show a positive *β*-catenin staining [8, 10].

Other treatments with antidepressant-like efficacy, such as the subchronic administration of SSRI fluoxetine together with the 5-HT_2A_ antagonist ketanserin, also produce a *β*-catenin increase in the membrane fraction but not in the nuclear one, which corresponds with a lack of changes in hippocampal proliferation (Figure 5(b)) [83]. The increase in membrane-associated *β*-catenin is parallel to an elevation of N-cadherin protein [83], both members of the *β*-catenin/N-cadherin complex present in pre- and postsynaptic terminals [223, 224], where *β*-catenin recruits scaffolding proteins [225], conforming cell-cell adhesion complexes [226], recruiting synaptic vesicles [209, 227], and acting on the development of new synapses [225]. This suggests a preference of modifications in synaptic plasticity instead of proliferation, as previously reported for other antidepressant treatments [60].

In addition, Frizzled receptors and GPCRs can interact through several pathways [228, 229]. Some GPCRs act through Gq and/or Gi proteins activating PKB (protein kinase B)/Akt which inhibits GSK-3*β* via phosphorylation. These receptors can also activate Gs proteins that activate prostaglandin E2 (PGE2), phosphoinositide 3-kinase (PI3K), and PKB/Akt, leading to the inhibition of GSK-3*β*. Other receptors act on Gq or G12/13 proteins, activating the phospholipase C*β* (PLC*β*) and protein kinase C (PKCs) and inhibiting GSK-3*β* [228]. Taken together, these data support the possible existence of interactions between the GSK-3*β*/*β*-catenin pathway and other neurotransmitter systems involved in depression, including serotonin. The pharmacological modulation of the different elements of the Wnt/*β*-catenin pathway with antidepressant purposes has to be clarified in the near future, probably modulating at the level of Wnts or *β*-catenin activity. Interestingly, a number of patents regarding GSK-3*β* inhibition as the therapeutic mechanism for treatment of neuropsychiatric disorders are being launched, including treatment of depression.

#### 3.3.3. mTOR Pathway

Target of rapamycin (TOR) genes, members of the phosphoinositol kinase-related kinase (PIKK) family of kinases [230], was first described in yeast as the pharmacological targets of the microbicide rapamycin [231]. TORs were subsequently described in other invertebrate and vertebrate organisms. mTOR, the mammalian form of this protein, exists in two different functional multiprotein complexes within the cells, mTORC1 and mTORC2, which are evolutionarily conserved from yeast to mammals [232, 233]. mTORC2 is involved in cytoskeletal remodeling [234] and in the regulation of cell survival and cell cycle progression. mTORC1, the primary target of rapamycin, is involved in cell proliferation, cell growth and survival by protein translation, energy regulation, and autophagy in response to growth factors, mitogens, nutrients, and stress [235–237].

In neurons, mTORC1 activity is regulated by phosphorylation in response to growth factors, as BDNF, mitogens, hormones, and neurotransmitters through the activation of G protein-coupled receptors (GPCRS) or ionotropic receptors. The mTORC1 phosphorylation is mediated by ERK/MAPK, PI3K, PKA, and Epac. The activation of mTORC1 results in the phosphorylation and activation of several downstream targets as the eukaryotic initiation factor 4E-binding protein 1 (4E-BP1), p70 ribosomal S6 kinase (p70S6K), RNA helicase cofactor eIF4A, extracellular signal-regulated kinase (ERK, including both ERK1 and ERK2), or PKB/Akt; and the inhibition of the eukaryotic elongation factor 2 kinase (eEF2) [238, 239].

mTOR has been extensively studied related to cancer, development, metabolism, and more recently to the central nervous system (CNS) physiology and diseases [238, 240, 241]. mTOR-signaling pathway is involved in synaptic plasticity, memory retention, neuroendocrine regulation associated with food intake and puberty, and modulation of neuronal repair following injury. The target proteins of mTOR, 4E-BP1, and eukaryotic initiation factor-4E (eIF4E) have been detected in cell bodies and dendrites in cultured hippocampal neurons and their distribution completely overlaps with the postsynaptic density protein-95 (PSD-95) at synaptic sites, suggesting the postsynaptic localization of these proteins [242]. The activation of mTOR has been functionally linked with local protein synthesis localized presynaptically as synapsin I, or postsynaptically as PSD-95 and GluR1, and cytoskeletal proteins as the activity-regulated cytoskeletal-associated protein (Arc) [27, 241, 243].

mTOR-signaling pathway has been also related to a number of neurological diseases, such as Alzheimer's disease, Parkinson's disease, and Huntington's disease, tuberous sclerosis, neurofibromatosis, fragile X syndrome, epilepsy, brain injury, and ischemic stroke [244]. Dysfunction of mTORC1 is associated with the pathogenic mechanisms of Alzheimer's disease, and the activation of p70S6K, downstream of mTORC1, has been identified as a contributor to hyperphosphorylated tau accumulation in neurons with neurofibrillary tangles [245].

Recent studies have also associated mTOR signaling in affective disorders since the administration of ketamine produces a fast-acting antidepressant-like effect in animals [27] and human [131]. In stressed rats, a reduction in PI3K-Akt-mTOR-signaling pathway has been reported in PFCx [127, 128] or amygdala [129]. The inhibition in mPFCx of calcineurin, a serine/threonine protein phosphatase that participates in the regulation of neurotransmission, neuronal structure and plasticity, and neuronal excitability, induces a depression-like behaviour [246], accompanied by a decrease in mTOR activity [130]. This effect can be reverted by the activation of mTOR by NMDA or the chronic administration of the antidepressant venlafaxine, promoting an antidepressant-like effect [130]. In human postmortem samples of prefrontal cortex of depressed subjects, there is a decrease in the expression of mTOR, as well as some of the downstream targets of this pathway, as p70S6 kinase (p70S6K), eIF4B, and its phosphorylated form, which suggests the impairment of the mTOR pathway in major depressive disorder (MDD) that would lead to a reduction in protein translation [247] (Table 2).

The subchronic, but not acute, administration of rapamycin in rodents has an antidepressant-like effect shown in two behavioural tests as forced swimming and tail suspension tests [248]. Acute administration of different NMDA receptor antagonists as the ketamine [27], Ro 25-6981 [27], and MK-801 [249] or antagonists of the group II of the metabotropic glutamate receptors (mGlu2/3), as MGS0039 and LY341495, produce a fast antidepressant effect [250, 251] mediated by mTOR-signaling pathway activation. Ketamine rapidly activates the mammalian target of rapamycin (mTOR) pathway, increases synaptogenesis, including increased density and function of spine synapses, in the prefrontal cortex of rats [27, 243], and increases hippocampal BDNF expression [252], that results in a rapid antidepressant-like effect in rats [27, 243] and humans [253]. Moreover, blockade of mTOR signalling by the specific antagonist rapamycin completely blocks the ketamine induction of synaptogenesis and behavioural responses in models of depression [27]. Other antidepressant strategies as the electroconvulsive treatment (ECT) also activate the mTOR pathway, leading to an increase in VEGF [254]. Therefore, modulation of mTOR could be a novel approach to develop strategies for the treatment of affective disorders [255].

## 4. A Further Step: Neuroplasticity versus Proliferation

The neurogenesis hypothesis of depression was based upon the demonstration that stress decreased adult neurogenesis in the hippocampus. This reduction in the production of newborn granule cells in the hippocampal dentate gyrus is related to the pathophysiology of depression. Since then, several studies have established that newborn neurons in the dentate gyrus are required for mediating some of the beneficial effects of antidepressant treatments since the increase in cell proliferation after antidepressant treatment is only observed in the SGZ and not in SVZ, suggesting a specificity of the antidepressants to regulate hippocampal neurogenesis. Moreover, psychotropic drugs without antidepressant activity do not increase neurogenesis [135, 256]. The disruption of hippocampal proliferation by irradiation is not sufficient to drive a depression-like phenotype. Both X-irradiation and genetic manipulation approaches demonstrated a requirement of hippocampal neurogenesis in mediating some of the antidepressant treatment effects [71, 76, 257], while mice exposed to X-irradiation of the SVZ or cerebellum responded normally to the antidepressants. However, some drugs with potential antidepressant action do not mediate their effect through the increase in hippocampal proliferation, as drugs acting on corticotrophin releasing factor receptor (CRF) or arginine vasopressin 1b (V1b) receptors [76], as indicated previously.

The appearance of the antidepressant-like effect in behavioural tests after 2-3 weeks parallels the time needed for the growth of newborn cells in hippocampus [258]. However, this time course does not always take so long. For classic antidepressants as the serotonin transporter inhibitors, a chronic regime is needed to observe that increased proliferation rate [10, 60], while, for others as ECS and 5-HT_4_ agonists [8, 64], an acute or subacute treatment, respectively, is enough to increase proliferation.

The putative role of changes in synaptic plasticity and/or neural proliferation in the depressive pathology is proposed some time ago [19]. Synaptic plasticity, as indicated for proliferation, is also modulated by antidepressant treatments [43, 44]. The neural plasticity is not only functional but structural and is impaired in animal models [259]. For example, there is a decrease in spine number in hippocampal CA1 and CA3 areas in bulbectomized animals that are reverted with antidepressant treatment [259–261]. This structural plasticity is more striking when new neurons are born [53], or there is an increase in neuron survival as a consequence of antidepressant treatment or ECS [262]. The new dendritic spines formed are associated to smaller postsynaptic densities (PSDs) and a higher frequency of miniexcitatory postsynaptic currents (mEPSCs), suggesting an increased number of new and active glutamatergic synapses [263].

The rapid antidepressant response to drugs as ketamine acting through the blockade of NMDA receptors appears as a new target for having fast-acting effects on the treatment of mood disorders compared to the weeks or months required for standard medications. Ketamine and other glutamate antagonists through the increase of the number and function of new spine synapses in rat prefrontal cortex by the activation of mTOR [27] do not modify hippocampal cell proliferation [264].

It would also be critical for future work to validate the relative importance of antidepressant-induced neurogenesis and synaptic plasticity in the antidepressant effects. However, evidence is strong that neurogenesis is required for at least some of the beneficial effects of antidepressant treatment. The exact role of neuroplastic/neuroproliferative changes in other brain structures as mPFCx and amygdala should be elucidated.

## 5. Conclusion

As indicated in this review, the importance of either proliferation or plasticity, or both, is still a matter of debate. As the involvement of proliferation and plasticity has been mainly studied in hippocampus, we might be underestimating its role in the antidepressant effect. In this sense, as the hippocampus is responsible for the learning and cognition part of the depressive disorder, the fact that the impairment of hippocampal proliferation would not block the antidepressant effect of some drugs does not necessarily conclude that the proliferation is only dependent on hippocampus. In the last years, prefrontal cortex, a structure with a great importance in mood control and working memory, is gaining increasing relevance in the plastic changes linked to antidepressant effects promoted by drugs as ketamine. In this sense, hippocampal proliferation would be only a small part of the plastic changes that are taking place within the hippocampus, and other brain areas. Thus, we must not underestimate the implication of synaptic plasticity in those antidepressant treatments that are not accompanied with increased proliferation.

## Figures and Tables

**Table 1:** Role of the activation and blockade of the different serotonin receptor subtypes in neural proliferation and synaptic plasticity.

Serotonin receptor subtypes	Effect of pharmacological manipulation on hippocampal proliferation				ko	
	Agonist		Antagonist			
	Change	Reference	Change	Reference	Change	Reference
5-HT_1A_	↑ proliferation (subchronic)	[77]	↓ proliferation (acute)	[78]	= proliferation	[9, 71]
			= proliferation (chronic)	[79]		
	↑ proliferation (chronic)	[74, 79, 80]	blocks 5-HT-induced proliferation	[78]	↓ cell survival	[9]

5-HT_2A/2C_	= proliferation (+SSRI)	[74]	↑ proliferation (chronic)	[81, 82]	*No data *	
			= proliferation (acute)	[74, 81]		
			↑ plasticity markers and BDNF (+SSRI, subchronic)	[83]		

5-HT_4_	↑ proliferation (subchronic)	[8, 64]	Blocks 5-HT-induced proliferation	[78]	*No data *	
	↑ plasticity markers and BDNF (subchronic)	[8, 63–65]				

5-HT_6_	* No data *		= proliferation	[84]	*No data *	
			↑ plasticity markers (PSA-NCAM)	[84]		

5-HT_7_	*No data *		↑ proliferation (subchronic)	[85]	= proliferation	[86]

↑: increase; ↓: decrease; ko: knock-out mice.

**Table 2:** Involvement of cAMP, Wnt/*β*-catenin, and mTOR-signaling pathways in major depression (MDD) and antidepressant treatment.

Signaling pathways		Changes related to disease or treatment						Direct effect	
		Nontreated MDD (versus control)			Treated MDD (versus untreated)				
		Change	Region	Reference	Change	Region	Reference	Change	Reference
cAMP/PKA	cAMP	↓ AC (induced by FK, *β*-AR, *α*2-AR)		[87–94]	↑ cAMP		[95, 96]		
					↓ 5-HT_1A_-induced AC inhibition/↓ 5-HT_4_-induced AC stimulation	Human PFCx	[8, 97, 98]	cAMP (administration/degradation inhibition) → AD-like effect	[99]
					↑ AC CB1-induced inhibition		[100]		
	PKA	*No data *			↑ PKA activity		[101]	*No data *	
	CREB	↓ CREB		[102–104]	↑ CREB (protein, expression, activity)	Hp, Cx	[11, 103–110]	CREB viral expression in Hp → AD-like effect	[44]
								CREB viral expression in Acb → depression-like effect	[111]
	Epac	↑ Epac-2	Hp, PFCx	[112]	*No data *			*No data *	

Wnt/*β*-catenin	GSK-3*β*	↓ GSK-3*β*	Hp	[113]	↑ GSK-3*β*	Hp	[8, 10, 113–117]	GSK-3*β* inhibition → AD-like and anxiolytic effect	[117–120]
								GSK-3*β* knock in → stress-induced depression	[121]
								GSK-3*β* SNP (−50 T/C; rs334558) → later onset of bipolar depression	[122]
								GSK-3*β* SNP (−50 T/C; rs334558) → ↑ antidepressant response	[123]
	*β*-catenin	↓*β*-catenin	PFCx	[124]	↑ *β*-catenin	Hp	[8, 10, 26, 83, 113–117]	*β*-catenin ko mice → depression-like effect	[125]
								*β*-catenin OE mice → AD-like effect	[126]

mTOR	mTOR	↓ mTOR, p70S6K, eIF4B, p-eIF4B		[127–129]	↑ p-mTOR/mTOR	PFCx	[27, 130]	Activation (NMDAr antagonists) → AD-like effect	[27, 131]

↑: increase; ↓: decrease; AC: adenylyl cyclase activity; FK: forskolin; Hp: hippocampus; PFCx: prefrontal cortex; Acb: accumbens nucleus; OE: overexpressing.
